# Comparison of the Pull-Out Resistance of Grossly Decayed Primary Anterior Teeth Restored With Two Different Intracanal Posts: An In Vitro Study

**DOI:** 10.7759/cureus.35643

**Published:** 2023-03-01

**Authors:** Ayham Hijaz, Mohamed K Altinawi, Imad Katbeh, Eyad Gergos, Gharawi Alhamzah

**Affiliations:** 1 Department of Pediatric Dentistry, Faculty of Dentistry, Damascus University, Damascus, SYR; 2 Department of Pediatric Dentistry and Orthodontics, Peoples’ Friendship University of Russia (RUDN University), Moscow, RUS

**Keywords:** fiber posts, pull-out resistance, glass fiber post, dentin post, primary anterior teeth, root canals, restorative dentistry, clinical dentistry

## Abstract

Background

Severely damaged primary anterior teeth that require pulp therapy present a high risk of failure due to the loss of tooth substance, resulting from pre-existing decay and endodontic therapy. The ideal post material should have physical and mechanical properties similar to those of dentin. Another concern in restoring endodontically treated primary teeth is the need to find a material that can resorb similar to the natural tooth structure as a part of the exfoliation process, allowing normal eruption of permanent successors. Accordingly, there is no such material other than dentin itself. The introduction of biological dentin posts offers an excellent alternative for restoring such teeth. This study aimed to assess the effect of using dentin posts on the pull-out resistance of endodontically treated primary anterior teeth in comparison to glass fiber posts.

Methodology

A sample of 30 primary anterior teeth was collected from the outpatient clinic of the Pediatric Dentistry Department, Faculty of Dentistry, Damascus University. A total of 15 freshly extracted permanent teeth with single roots were also collected from the outpatient clinic of the Maxillofacial Surgery Department, Faculty of Dentistry, Damascus University. The roots of the permanent teeth were used to prepare 30 dentin posts using a CAD-CAM machine. After receiving proper endodontic treatment, the primary teeth were divided into two groups (15 teeth in each group). The first group was restored with dentin posts, and the second was restored with glass fiber posts, with the posts measuring 3 mm in length for both groups. Pull-out resistance testing was performed using a Testometric machine.

Results

The arithmetic mean of the forces applied in the glass fiber posts group was 153.2 ± 39.12 N, while the arithmetic mean of the forces applied in the dentin posts group was 156.7 ± 39.78 N. The data were analyzed at a 95% confidence interval using the independent Student’s t-test. There were no statistically significant differences in pull-out resistance between the two groups.

Conclusions

Dentin posts showed a little increase in pull-out resistance than glass fiber posts. Therefore, the use of dentin posts as intracanal retention in primary anterior teeth is a successful alternative for composite posts.

## Introduction

Early childhood caries is one of the most common dental and oral problems in children and is defined as the presence of one or more teeth affected by caries at the age of less than three years [1]. Early childhood caries is characterized as a rapidly developing necrotic process that begins in the cervical third of the primary maxillary incisors shortly after their eruption and continues to worsen until it includes the whole crown. If untreated, it can lead to early tooth loss [2]. It appears, as mentioned earlier, to first affect the primary maxillary incisors but then involves the primary first upper and lower molars, and, lastly, the primary canines in both jaws. Advanced caries of teeth results in the child experiencing severe pain, which negatively affects their nutrition and the ability to eat and drink. This, in turn, causes a lack of nutrition in the child and the concomitant lack of growth and delay. As the problem continues to worsen, the affected teeth die, causing peak lesions that are foci of infection in the child’s oral cavity [3].

From an orthodontic viewpoint, the premature loss of primary anterior teeth at the age of less than three years results in perimeter impairment of the anterior dental arch, in addition to an increase in the likelihood of developing abnormal oral habits, such as finger sucking or tongue thrusting, and thus the development of subsequent malocclusion [4]. Another negative consequence associated with the early loss of upper primary anterior teeth is the incorrect development of speech in a child, which may be permanent if it occurs early and remains untreated [5].

At the psychological and social levels, early childhood caries causes psychological and behavioral problems in children due to unattractive appearance, which weakens their self-confidence and makes them shy and introverted children who avoid contact with others for fear of being bullied [4].

Treating early childhood caries is problematic and requires cooperation between families and dentists [6]. Moreover, treatment relates to several factors, such as the extent of the lesion, the age of the child and their behavior, and the degree of parents’ awareness and understanding [7]. The first step in addressing caries is a time-consuming one. It involves making the parents aware and monitoring the oral and dietary habits of the child to identify harmful habits and work toward getting rid of them. This is followed by reconstructive dental treatments, where the initial necrotic lesions are treated by applying glass ionomer cement or compomer resins after removing the softening dental tissue using hand tools [6]. In the case of deep caries involving damage to the pulp, the treatment is through endodontic treatment and the use of short posts to provide the necessary support for coronal restoration [8]. Various forms of posts have been proposed to be used as endodontic retainers when restoring primary anterior teeth, including reversed-orientated metal posts [9]; orthodontic wires of various shapes such as omega-shaped (Ω) [10], alpha-shaped (α) [11], or gamma-shaped (γ); wire posts [12]; and carbon fiber posts [13]. More aesthetic alternatives such as composite resin posts [14], polyethylene posts [15], or glass fiber posts have also been used [16]. These options are characterized by several positive features such as corrosion resistance and biocompatibility, in addition to their desirable mechanical properties. However, these posts cannot simulate the biological characteristics of the dental structure of temporary teeth, represented by the absorbability of this structure in a way that allows the natural emergence of permanent teeth [17].

Dentin posts are a promising alternative that can be used in the restoration of severely damaged primary anterior teeth having appropriate characteristics, as these posts can theoretically be absorbed naturally as part of the physiological process of primary teeth loss and replaced with a permanent successor. This can be considered a special advantage of these posts, making them the ideal option for the rehabilitation of severely damaged temporary anterior teeth in pediatric dentistry. It is expected that these posts will have physical properties similar to those of teeth such as modulus of elasticity, compressive strength, and thermal expansion, among others [18]. This study aimed to compare the pull-out resistance of dentin and glass fiber posts used in the restoration of severely decayed anterior teeth.

## Materials and methods

This study was conducted at the Department of Pediatric Dentistry, Faculty of Dentistry, Damascus University. The sample size was calculated using G*Power 3.1.9.7 computer program. A minimum sample of 10 was set to ensure that an adequate sample size was collected at 95% power, an effect size of 0.8, and a 5% level of significance. The study sample consisted of 30 primary anterior teeth (maxillary incisors and canines, as well as mandibular canines) collected from patients attending pediatric dental clinics, where the tooth was required to be included in the sample to meet the following conditions: The presence of at least two-thirds of the root length. The root must be intact and free of caries, fractures, or cracks. The tooth extraction was caused by traumatic injuries or necrosis of early childhood caries that led to a significant destruction of the crown with the refusal of parents for treatment or the extraction of primary maxillary or mandibular canines for orthodontic purposes. The exclusion criteria were as follows: The tooth had undergone previous endodontic treatment. The roots showed any sign of the presence of internal and external resorption, which could be confirmed radiographically.

The study protocol was approved by the Scientific Research and Postgraduate Board of Damascus University Ethics Committee, Damascus, Syria (IRB number: UDDS-568-22072019/SRC-3674).

Preparation and filling of primary anterior teeth

After the sample collection was completed, the samples were stored within the serum until use. The crown was cut 1 mm using cylinder bur (sf-41) above the cementoenamel junction perpendicular to the longitudinal axis of the tooth in an attempt to simulate clinical decay of teeth.

The initial working length of the root was determined by macroscopic examination judging by the emergence of a 10-gauge K-file from the root apex. Preparation was done using a Hedstrom file, up to 40 mm in size, for the entire working length with irrigation using sodium hypochlorite at a concentration of 5.25%. After preparation and irrigation, the canal was dried using absorbent paper points and then filled with Metapex material using a Lentulo spiral into the canal.

In total, 30 anterior primary teeth were divided into two groups of 15 teeth (the first group was treated with dentin posts, and the second was treated with glass fiber posts). Seven posts measuring 1.4 mm in diameter and eight posts measuring 1.2 mm in diameter were used in each group.

Selection of glass fiber posts

In this study, 15 glass fiber posts of the DenMat type (Den-Mat, Santa Maria, CA) with a diameter of 1.2 or 1.4 mm were used, which are available in the form of sets, with each set including two packages containing five posts each. These posts were chosen because they do not need any pretreatment of the post surface before application (according to the manufacturer’s instructions), and the preparation of the post space can be accomplished using Gates-Glidden drills (Mani Gates, Mani Inc, Tochigi, Japan), which are available in several diameters (we used 1.2 mm and 1.4 mm), providing flexibility in choosing the suitable post.

Preparation of dentin posts

A total of 15 permanent teeth with a single root and a single canal were collected from the Department of Oral and Maxillofacial Surgery, Faculty of Dentistry, Damascus University. The criteria for inclusion were a single root and a single canal (confirmed radiographically), straight without curvatures, freshly extracted, fully developed apices, intact and free of caries, cracking or fractures, and removed for orthodontic purposes or due to periapical lesions.

**Figure 1:**
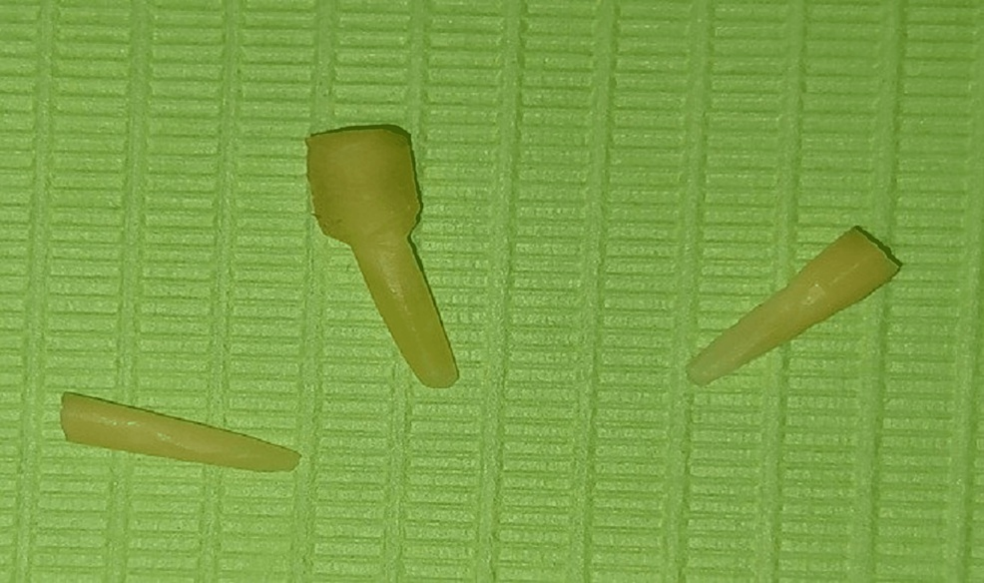
Prepared dentin posts.

After collecting the teeth matching the above criteria, the crown was separated from the root below the cementoenamel junction using a high-speed turbine and sf-41 diamond bur with a 1 mm diameter. Following this, the root was split in half to ensure that the root canal was not located within the post structure after preparation. The dentin posts were then threaded with dimensions similar to the dimensions of the glass fiber posts prepared for use in the study (15 posts with a diameter of 1.2 mm and 15 posts with a diameter of 1.4 mm) and a length not less than 8 mm using a CAD-CAM device (DWX-52 Milling Machine, Roland, Japan) (Figure 1).

Finally, the posts were sterilized by immersing them for two weeks in a 10% formalin solution, according to the protocol approved by the Centers for Disease Control and Prevention, and then washed and stored within the serum until use [19].

Preparing the post space

The post space was prepared using Gates-Glidden drills (Mani Gates, Mani Inc, Tochigi, Japan) up to the fourth size of 1.1 mm when using a post with a diameter of 1.2 mm and up to the fifth size of 1.3 mm when using a post with a diameter of 1.4 mm (according to the instructions of the manufacturer of glass fiber posts used in the current study, i.e., DenMat). A rubber stopper was placed along a length of 4 mm, which ensured a 3 mm extension of the post below the cementoenamel junction because the height of the remaining ferrule was 1 mm above the cementoenamel junction.

Post-cementation and coronal buildup

The posts were fixed by pursuing the following steps in each studied group:

Glass Fiber Posts Group

The post was wiped with a cotton roll moistened with alcohol and dried without etching or bonding according to the manufacturer’s instructions.

The prepared post space was then cleaned and acid etched with 37% phosphoric acid (Meta-Biomed Co. Ltd, Korea) for 15 seconds, and the coronal enamel was etched for 30 seconds, followed by rinsing with water and air drying for 10 seconds. The bonding agent Tetric N-Bond (Ivoclar Vivadent, Schaan, Lichtenstein) was brushed on the etched surface and post and light-cured using Woodpecker DTE LUX E Plus for 20 seconds. Lastly, the dual-cure resin cement Variolink II (Ivoclar Vivadent, Schaan, Lichtenstein), where an equal amount of the two components of the cement were mixed well, was inserted into the canal chamber with a Lentulo spiral, after which the post was inserted and cured for 40 seconds. Further, the post was cut to a height of 3 mm above the cementoenamel junction.

Dentin Posts Group

After ensuring that the walls of the post space were clean, etching was done using 37% phosphoric acid (Meta-Biomed Co. Ltd, Korea) for 15 seconds for each of the dentin posts and post spaces. The coronal enamel was then etched for 30 seconds, rinsed with water, and air-dried following the same protocol as in the first group, applying the bonding agent Tetric N-Bond (Ivoclar Vivadent, Schaan, Lichtenstein) on the etched surface and the post and light-cured for 20 seconds, followed by using dual-cured resin cement Variolink II type (Ivoclar Vivadent, Schaan, Lichtenstein). The post space was filled as in the first group, and the post was inserted into dual-cure resin cement and cured for 40 seconds, after which the post was cut to a height of 3 mm above the cementoenamel junction.

The core buildup was done using Tetric N-Ceram composite resin (Ivoclar Vivadent, Schaan, Lichtenstein) layer by layer until the crown construction was complete and provided the appropriate anatomical shape, achieving a crown 4 mm above the cementoenamel junction.

Thermocycling

After completing the restoration, the study samples underwent 500 thermal cycles. One cycle included inserting the sample into a warm water bath at 55 ± 1°C, followed by a cold water bath at 5 ± 1°C. The sample was left for 30 seconds in each of the baths with an interval of five seconds between a warm and cold bath in an attempt to simulate the condition of the oral environment.

Withdrawal tests

**Figure 2:**
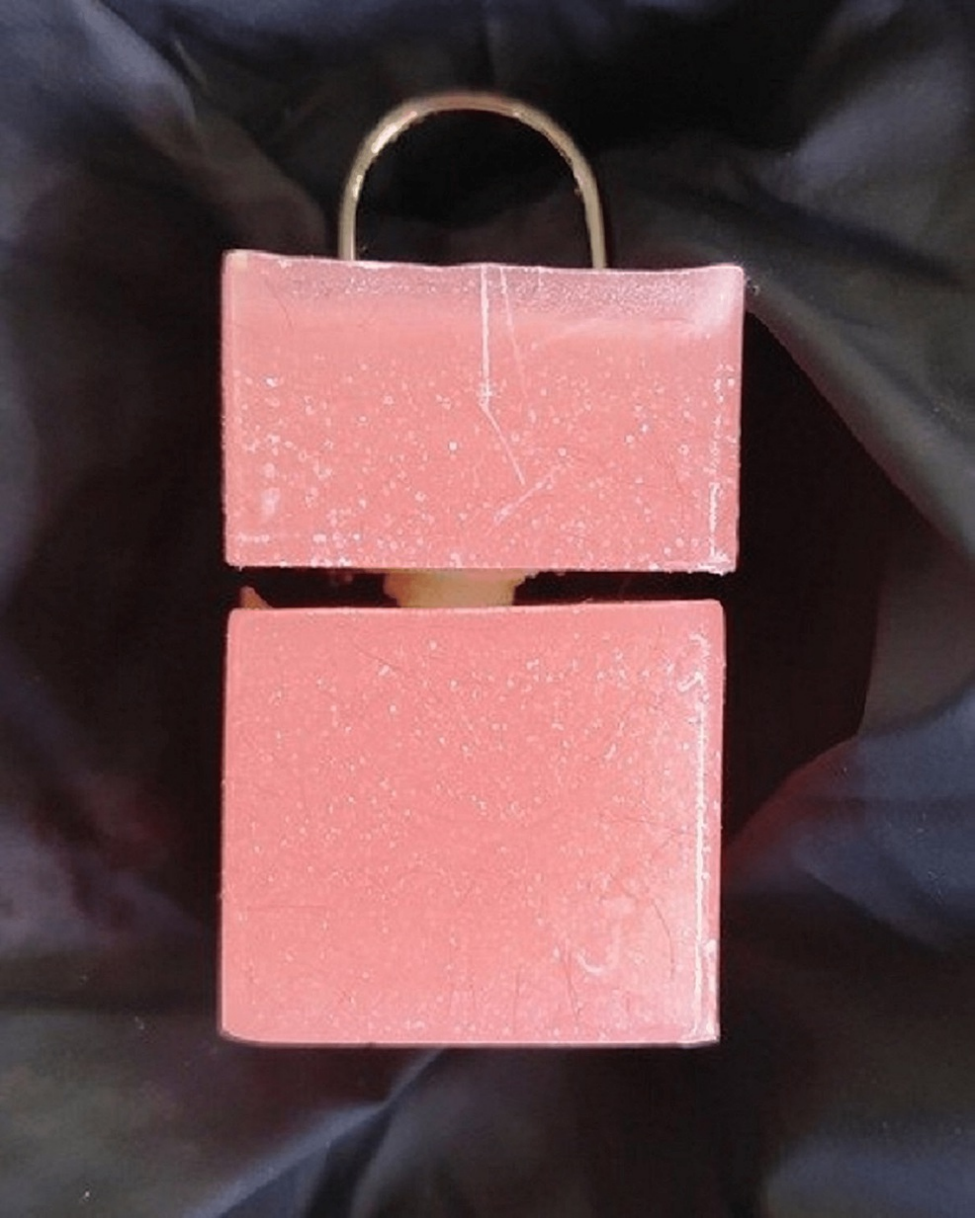
The tooth after being placed within the acrylic models.

After the restoration and thermocycling of the study sample were completed, a heavy bur with a diameter of 0.6 mm was used to drill holes into both the root and the restored crown with composite to secure the necessary stability of the tooth within the completed acrylic model. The tooth was placed within two acrylic models, where the first covered the root up to 1 mm below the cementoenamel junction, while the second covered the completed restoration without including the remaining ferrule, i.e., its boundaries were from the top of the coronal restoration up to 1 mm above the cementoenamel junction. The addition of a loop of orthodontic wire with a diameter of 1 mm at the apex of the coronary Acryl was done in such a way as to allow the push-out bonding strength test to be performed correctly (Figure 2).

During the placement of the tooth within the acrylic molds, it was placed strictly perpendicular to the acrylic bases so that the pulling forces were applied according to the longitudinal axis of the tooth and the used post.

**Figure 3:**
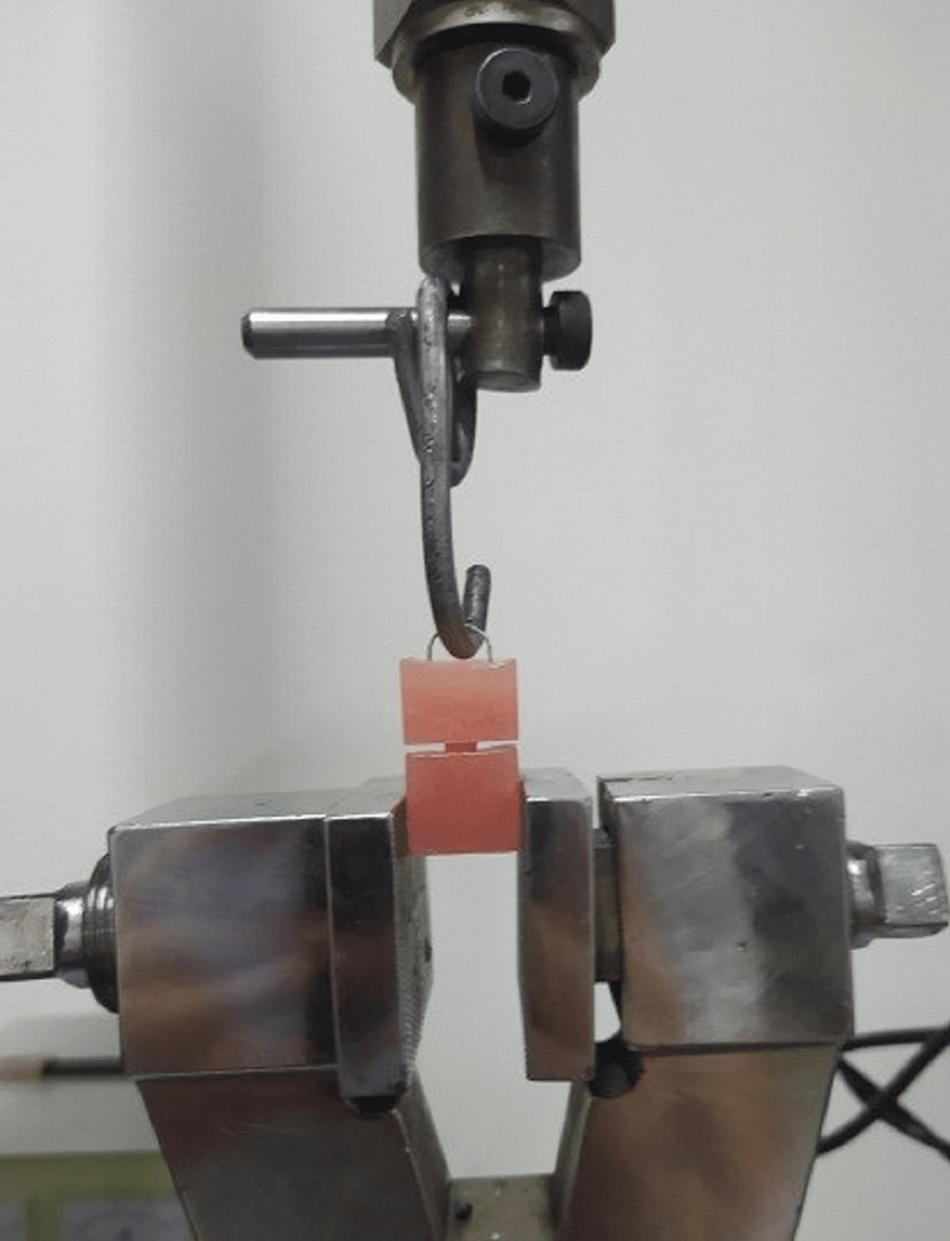
Performing the test on a sample.

The pull-out test was performed using the general mechanical Testometric testing device at a speed of 0.5 mm/minute (Figure 3), and, in the event of a failure, the applied force estimated in newtons (N) was recorded. Moreover, the failure pattern obtained by macroscopic examination was recorded, which was divided into the following three patterns: (i) Separation at the level of the post-cement; (ii) Separation at the level of cement-canal walls; and (iii) Separation of the restoration from the post with the post remaining within the root canal.

The results were recorded, and statistical tests (Kolmogorov-Smirnov, Student’s t-test, and chi-square test) were performed using SPSS version 24 (IBM Corp., Armonk, NY, USA), with the adoption of the null hypothesis based on the absence of statistically significant differences between the two groups in terms of their resistance to displacement.

## Results

**Figure 4:**
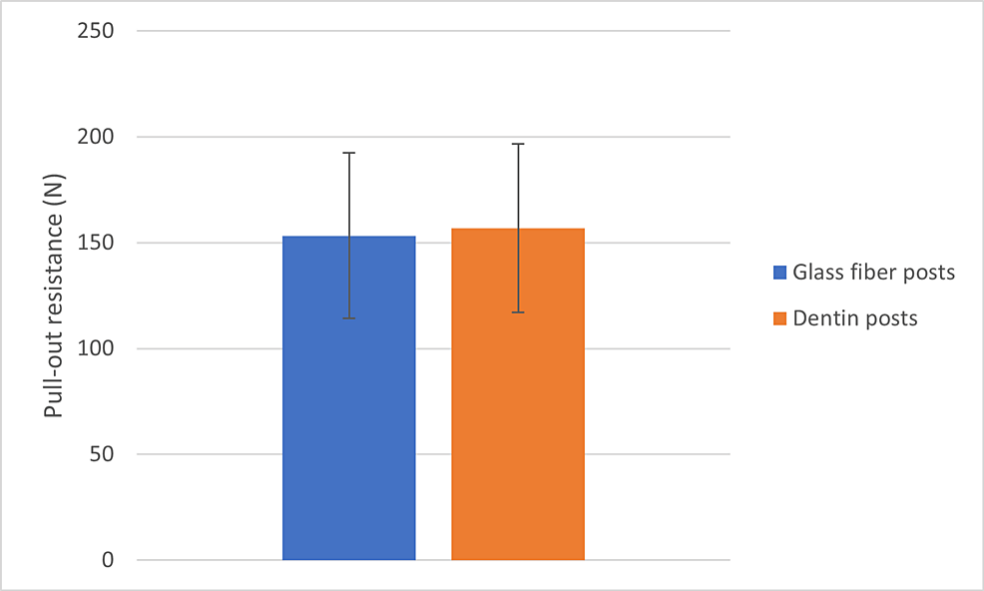
The differences between the calculated averages of the pull-out resistance variable in the two groups of the research sample (N: Newton).

The results were collected, and the arithmetic mean of the forces at which failure occurred in each group was calculated. The arithmetic means of the forces applied in the glass fiber posts group was 153.2 ± 39.12 N while that in the dentin posts group was 156.7 ± 39.78 N, as shown in Figure 4.

The Kolmogorov-Smirnov test was performed to investigate the distribution pattern of the applied forces in each of the two study groups. The p-value was >0.05, so the data had a normal distribution. Based on this result, the parametric statistical tests were chosen to compare the pull resistance between the two study groups with the Student’s t-test for independent samples.

The Student’s t-test was conducted for independent samples to study the differences between the average forces applied in the two groups, and the value of the significance level was (at 95% confidence range) 0.811, which reflects the absence of a statistically significant difference between the average forces applied in both groups.

The frequencies and percentages of failure patterns in both groups were also recorded, as shown in Table 1. The cement-post failure pattern dominated both groups followed by failures at the level of cement-canal walls, and the restoration-post failure pattern was expressed only in one case using a glass fiber post.

**Table 1:** Percentages of repetitions of failure patterns in two groups of the research sample.

Failure patterns	The studied group					
	Percentages			Frequencies of repetition		
	Glass fiber posts	Dentin posts	Total	Glass fiber posts	Dentin posts	Total
Post-cement	66.7%	80%	73.3%	10	12	22
Cement-canal walls	26.7%	20%	23.3%	4	3	7
Restoration-post	6.7%	0%	3.3%	1	0	1
Total	100%	100%	100%	15	15	30

Finally, the chi-square test was performed to study the differences in failure patterns between the two study groups, and there were no significant differences (p = 0.516).

## Discussion

Maintaining the primary occlusion in proper condition is an important factor in the development of the child. It not only ensures the safety of function and pronunciation, in addition to securing the aesthetic aspect and keeping space for permanent successors, but also prevents the development of abnormal oral habits [20].

One of the common challenges in pediatric dentistry clinics is the restoration of severely decayed primary anterior teeth, which are usually seen in children with early childhood caries or those who have suffered traumatic injuries that led to the loss of the coronal structure of these teeth. These cases are accompanied by a decrease in proper mastication and loss of vertical dimension, in addition to the possibility of developing non-functional habits, such as tongue thrusting and oral breathing. In the future, this leads to the development of malocclusion and impacts aesthetics and phonetics, potentially even affecting the child’s personality and behavioral development [21].

The restoration of severely decayed primary anterior teeth has always been a major challenge for pediatric dentists due to several factors, the most prominent being the age at which these injuries occur, usually associated with difficulties in the behavioral management of the child. In addition, the small size of the crown and the widening of the size of the pulp chamber, and, therefore, the insufficiency of the remaining healthy dental structure, also lead to issues [22]. These difficulties are also heightened because of the structural differences in primary teeth compared to permanent teeth because the former are unable to provide bonding forces such as those provided by permanent teeth [23].

Composite has always been the most commonly used option for the restoration of these teeth due to its aesthetics and wear resistance [24]. However, the need to achieve additional retention of the core of endodontically treated teeth, especially when there is a significant loss of dental tissue, led to the use of intracanal retainers [25]. Among the multiple options available at the level of posts used in the restoration of primary anterior teeth, two types of posts were adopted in this study. Glass fiber posts were chosen due to their positive properties, such as aesthetics, bio-acceptability, and wear resistance, in addition to their mechanical properties similar to dentin [26]. They have also been used to support weak roots without increasing the likelihood of root fracture, along with their ease of application and installation, which improves the connection with both the luting agent and the restorative material used to build the core above it [27]. Dentin posts have also been used as a promising alternative for the restoration of primary anterior teeth because they have the unique basic feature of absorbability, allowing for a smooth replacement of primary teeth and the eruption of their permanent successors [11,21].

Restoration fracture or displacement are the two main causes of failure after the rehabilitation of severely worn primary anterior teeth. Therefore, stability and resistance to pulling forces are desirable for any restoration [28].

There are several mechanical tests used to assess the retentive ability of adhesive posts, such as the push-out test, the microtensile technique, and the pull-out test, each of which has its own features; however, the current study adopted the pull-out test because it allows a simulation closer to clinical reality, where the stability of the post is studied as a whole unit along the entire length of the root canal [29].

The results of this study did not show any significant differences between the two groups in terms of binding strength, which averaged 156.7 N in the dentin posts group and 153.2 N in the glass fiber posts group. This is consistent with several studies, such as the study by Pinheiro et al., which concluded that there were no significant differences between dentin posts, short composite posts, and α-shaped wire loop, although the dentin posts group had the highest average [11]. The same was reported by Pithan et al., which did not find significant differences in the following three posts used for the restoration of severely decayed lower anterior teeth: short composite posts, γ-wire posts, and glass fiber posts [30]. The study by Gujjar and Indushekar showed the superiority of glass fiber posts over both orthodontic gamma-wire posts and short composite posts with statistically significant differences [26]. Chapla et al. also compared dentin posts and glass fiber posts and found that teeth restored with dentin posts exhibited marginally better fracture resistance than those restored with glass fiber posts [17]. The discrepancy between the studies can be explained by the different types of posts used, the difference in the level of adhesive cement, and the adhesion protocol used, in addition to the different teeth used to prepare dentin posts and the different lengths and diameters of these posts. A study by Memarpour et al. also showed a statistically significant superiority of composite posts over the rest of the groups, which can be explained by the form of the selected preparation of the post space of composite posts. This contributed to securing additional mechanical stability and resistance to their displacement outside their post space when applying pulling forces [28].

Regarding the failure patterns occurring, the failure of the connection at the post-cement level was predominant in both groups, where it appeared by 80% in the dentin posts group. This can be explained by the fact that both the post and the walls of the canal are made of dentin, and, therefore, the bond between them and the adhesive cement is the same. However, the contact surface between the walls of the canal and the adhesive cement is larger than that between the post and the cement, which leads to a stronger bond between the cement and the walls of the canal and thus the failure at the post-cement level.

The failure also dominated the post-cement level in the group of glass fiber posts by 66.7%, which can be explained by the fact that the highly cross-linked polymer matrix of these posts makes it difficult for the effective free monomers in the adhesive cement to chemically bond with the post structure. This results in a mechanical bond between the post and the adhesive cement, and, therefore, we return to the situation where the contact surface between the walls of the canal and the adhesive cement is larger than that between the post and the adhesive cement with a symmetrical bond on both sides. Thus, bond failure occurs at the post-cement level. This study agrees in terms of the prevailing failure pattern with several previously reported studies [6,11,26], while the current results differed from other studies [28], where the prevailing failure pattern was at the level of cement-canal walls. The difference can be attributed to the method used in applying adhesive cement, where the adhesive cement is mixed and then the post is placed within the resulting mixture to cover it with adhesive cement and then applied within the canal, which causes the creation of air bubbles between the canal walls and the cement. The difference can also be attributed to the use of zinc oxide and eugenol as a filler for the root canal. The presence of any residue of this substance on the dentin walls of the post space would compromise the uniformity of the adhesive cement as a resinous material.

The failure pattern of cement-canal walls also appeared in both groups: by 20% in the dentin posts group, and by 26.7% in the glass fiber posts group. The failure of this pattern can be explained by insufficient drying of the dentin walls of the post space after acid etching and washing, which harms the bonding agent with these walls. This failure can also result from the formation of air bubbles between the adhesive cement and the walls of the post space, which weakens the bonding strength obtained at the level of the cement wall. These bubbles can form during the mixing of the two components of the adhesive cement or during the introduction of cement to the post space [28]. Moreover, this pattern of failure can also be caused by the high value of the C factor and the resulting unavoidable polymerization shrinkage, which weakens the bond of cement with the dentin walls of the post space [28].

There was only one failed sample in the group of glass fiber posts (6.7%), which may have been a result of moisture contamination between the layers of the composite during the application of the coronary restoration or the presence of an air bubble between two layers of the composite during application.

The extraction cases in orthodontics are increasingly undesirable, which led to more difficulties in obtaining the dentin posts necessary to ensure adequate sample size. The small sample size was due to the difficulty in finding permanent teeth indicated for extraction posed as an added limitation to this laboratory study. Furthermore, it is difficult to reproduce intraoral conditions during in vitro studies, even though we thermally cycled the samples to somewhat mimic the clinical conditions of the oral cavity. The periodontal effect was not reproduced in this study and all roots were cast directly into acrylic blocks.

Considering these limitations of the study, we emphasize the importance of future clinical studies with a larger sample size to support the results of this laboratory study.

## Conclusions

Based on the results of this study, it can be concluded that dentin posts in terms of their pull-out resistance showed a slightly higher average. In addition, the absorbability of dentin posts makes them a preferred option for the restoration of severely damaged primary anterior teeth.
